# Reliability of tachycardia as an early warning sign in acute clozapine-induced cardiac inflammation: Protocol for a systematic review

**DOI:** 10.1371/journal.pone.0341729

**Published:** 2026-02-25

**Authors:** James Richard O’Neill, Prakruti Babu Narendra Prasad, Matthew Croft, Matthew Manton, Natalie King, Daniel Romeu

**Affiliations:** 1 University of Leeds, Leeds, United Kingdom; 2 South West Yorkshire Partnership NHS Foundation Trust, Wakefield, United Kingdom; 3 Tees, Esk and Wear Valleys NHS Foundation Trust, Harrogate, United Kingdom; Cholistan University of Veterinary and Animal Sciences, PAKISTAN

## Abstract

**Introduction:**

Clozapine-induced cardiac inflammation (CICI) can have serious consequences for people with psychosis and can be fatal if not detected in a timely manner. The best available methods of investigation for CICI are resource-intensive, whereas clinical practice often utilises cheaper and more practical methods of detection such as history, examination, and blood tests. Heart rate monitoring is commonly used as an early-warning sign in detecting potential CICI. Previous studies have found that, although this has a high sensitivity, it has poor specificity. As far as we are aware, no systematic review has investigated the role of tachycardia as a detection tool for CICI.

**Methods and analysis:**

Systematic diagnostic test accuracy review of the available literature will be used to assess the effectiveness of tachycardia as an early-warning sign for CICI. We will search eight electronic databases and grey literature using a comprehensive and systematic approach. Grey literature sources will include conference proceedings and professional guidance documents identified through expert consultations. Retrieved articles will be critically appraised, synthesised, and meta-analysed, where data permit.

**Ethics and dissemination:**

This systematic review will use data from existing studies, hence no ethical approvals are required. Findings will be disseminated to academic colleagues through a peer-reviewed publication and relevant academic meetings, and to clinicians through distribution to local and national clinical networks.

PROSPERO registration ID: CRD42025650379

## Background

Myocarditis is the inflammation of, and subsequent damage to, the heart muscle [1]. Pericarditis is also an inflammatory process which affects the pericardium layer around the heart muscle [2]. Whilst pericarditis has a mortality rate of around 1.1% [3], myocarditis has a much higher mortality rate of between 21–50% [4].

Both conditions have wide-ranging aetiologies, encompassing infection, toxins, and hypersensitivity [1,5]. Cardiac inflammation can sometimes be iatrogenic in nature through adverse drug reactions, for example when initiating clozapine. Clozapine is the only licensed medication for treatment-resistant psychosis [6]. However, it is often underutilised in clinical practice, in part due to its associated adverse drug reactions [7,8]. Initiation must be implemented in a slow and gradual manner to avoid various adverse effects such as pneumonia [9] and myocarditis [10].

Cardiac inflammation affects 1–3% of people prescribed clozapine [11,12]. Clozapine-induced cardiac inflammation (CICI) results from an IgE-mediated hypersensitivity reaction [13], and often occurs in the first few weeks of treatment [14]. CICI is more likely to occur if titration is undertaken rapidly [15,16], and the risk can therefore be reduced if titration is slowed [17,18]. The risk of CICI has also been observed to increase if clozapine is prescribed alongside either sodium valproate [19] or selective serotonin reuptake inhibitors [20].

Timely detection of CICI is fundamental given its high mortality rate [4,21], but a practical and reliable test is currently unavailable, particularly within a psychiatric setting. The best available method for diagnosis of myocarditis is endomyocardial biopsy [22], but this is highly invasive, resource-intensive, and often impractical. Cardiac magnetic resonance imaging (MRI) has been found to have a higher sensitivity than echocardiograms (48%) or troponin I testing (39%), but even still, cardiac MRI fails to detect one in every four cases of CICI [23].

Early clinical recognition of CICI improves outcomes; it is managed through prompt cessation of clozapine and commencing supportive treatment. However, the value of clinical factors alone in conclusively diagnosing CICI is questionable [24]. This is due to wide-ranging clinical presentations and often poor diagnostic value.

Tachycardia and fever have previously been found to have the highest sensitivity of clinical signs and symptoms [25] from a study of 75 cases. Heart rate greater than 100bpm detected around 90% of cases of myocarditis, whilst a body temperature greater than 38 degrees Celsius detected almost 80% of cases.

Tachycardia can also manifest as a benign side-effect from clozapine treatment [26], and elevated heart rate has been found to have a specificity value of less than 10% [27]. A fever of 38 degrees Celsius, however, has been found to have a specificity of over 90% for myocarditis [25]. Fever in clozapine treatment can be caused by various aetiologies, many of which are benign and would not indicate cessation of the drug [28].

Through this review, we aim to synthesise research on CICI to assist clinicians in managing this fatal condition, building on previous work in this area [25] by synthesising other published cases in literature.

### Aims

This systematic diagnostic test accuracy review aims to assess the effectiveness of tachycardia as an early-warning sign for CICI. The primary aim is to determine the sensitivity of tachycardia in confirmed cases of CICI by reviewing case studies, case series, and observational studies. Secondary aims include reviewing the clinical utility of other signs and symptoms associated with CICI, such as chest pain and shortness of breath, as well as the sensitivity of various blood markers proposed to investigate CICI including C-reactive protein, troponin, and white cell count.

## Methods and design

### Population

This review will include all adults (aged 18 and over) who have been prescribed clozapine. Both inpatient and outpatient clozapine titration in both primary and secondary care settings will be included. However, it is highly likely that clozapine would be initiated within a specialist psychiatric service rather than primary care or a general hospital setting. Patients with underlying cardiac conditions, such as coronary heart disease and heart failure, will not be excluded from analysis.

### Index test

We will observe the reported changes to heart rate that occur during the titration period, and whether tachycardia occurred. We have defined tachycardia as either being 100 beats per minute (bpm) or higher, or a rise of 30bpm from the pre-clozapine baseline. Both thresholds (>100 bpm or 30 bpm from baseline) will be recorded for each case where available, allowing assessment of concordance between definitions.

### Reference tests

We will compare the sensitivity of tachycardia to the best available method to identify CICI which is endomyocardial biopsy. However, due to limited availability of such a test, we will also use various other signs and symptoms associated with CICI as reference tests. These will include confirmation of diagnosis via imaging techniques such as magnetic resonance imaging (MRI) or echocardiogram.

### Diagnosis of interest

We will include cases of cardiac inflammation (consisting of myocarditis, pericarditis, myopericarditis, or perimyocarditis) which occur within 45 days of a person commencing clozapine treatment. Any cases occurring after this point will be excluded; this figure of 45 days has been chosen due to the low chance of cardiac inflammation after this point being related to the clozapine titration process [25]. We will also exclude any cases of cardiomyopathy not involving inflammatory process, as well as any reported cases of CICI which were not confirmed by biopsy or imaging techniques (cardiac MRI or echocardiogram). This standard has been used as an alternative reference test [22] due to the rare use of endomyocardial biopsy in clinical practice.

### Study design

We will collate existing published case reports, case series and observational studies (both retrospective and prospective cohort studies, case-control, and cross-sectional studies) of CICI. Randomised controlled trials or interventional studies are not applicable for the nature of this study and will therefore be excluded.

We envisage that the available literature in this regard will predominantly take the form of a basic test accuracy study design, whereby both heart rate and reference tests are undertaken at the same time or in close succession [29].

### Search strategy

We will search international literature through eight electronic databases relating to medicine, psychology, and health (Ovid MEDLINE, Ovid EMBASE, Clarivate Web of Science, EBSCO CINAHL, APA PsycINFO, Scopus, Ovid International Pharmaceutical Abstracts, and the Cochrane Library). We will search from database inception to present day in order to fully explore the variety of clinical applications of clozapine over the years since its discovery in 1958 [30].

Our search strategy has been devised with the expertise of a wide range of professionals, both clinical and academic. This includes clinical and academic psychiatrists (JO, DR, MM), mental health pharmacists (MC), a medical student (PP), and an information specialist (NK). Our preliminary strategy for Ovid MEDLINE is listed in S1 Fig. Our other seven strategies have been devised with the assistance of Polyglot software [31] and peer reviewed by an information specialist (NK).

We will scan the reference list of included studies and use forward citation searching (seeing which papers have cited any relevant results from our search) by one generation. We will also contact experts in the field, such as key authors, in order to maximise the likelihood of finding all relevant results. We will re-contact them after four weeks if no response is received from an initial e-mail. Finally, a supplementary grey literature search strategy of targeted websites and key guidelines (such as UK and international college guidelines and databases for drug repositories) will be developed through consultations with experts and expanded iteratively in view of retrieved articles. Clinical trial registers will not be included in this review due to the nature of CICI epidemiology. However, conference abstracts were reviewed and considered for inclusion.

Articles written in other languages to English will be included and use of Google Translate software will be employed to assist in translation. This approach has been chosen due to the largely quantitative focus of this review, and the fact that slight nuances that may be misrepresented through translation would not be felt to significantly affect the interpretation of results.

### Eligibility screening and study selection

One reviewer (JO) will perform the search strategies (see S1 Fig) and de-duplicate records using Clarivate EndNote software [32]. Two reviewers (JO & PP) will independently screen articles using Rayyan software [33] based on eligibility criteria (see Table 1 and S2 Fig). Any discrepancies or differences in opinion will be resolved by consensus and discussion with a third unblinded reviewer (DR). Pilot screening was not deemed to be necessary due to the comprehensive approach undertaken during our screening process.

**Table 1:** Inclusion and exclusion criteria checklist based on the PRISMA guidelines [34] (see S2 Fig) but with alternative PIRD framework [35,36].

	Inclusion	Exclusion
**Study designs**	Case reports Case series Cohort studies (retrospective and prospective) Case-control studies Cross-sectional studies	Randomised controlled trials
**Population**	Adults aged 18 and over Commenced on clozapine within 45 days of symptom onset	Children/adolescents Established on clozapine >45 days before onset of symptoms
**Index test**	Heart rate elevation (defined as >100bpm or increase of >30bpm from pre-clozapine baseline)	Heart rate not recorded
**Reference tests**	Endomyocardial biopsy Cardiac imaging (MRI or echocardiogram)	
**Target condition/ diagnosis of interest**	Myocarditis, pericarditis, or combination of both Occurring within 45 days of clozapine titration commencing	Cardiomyopathy of non-inflammatory nature Endocarditis Symptoms developing after 45 days of clozapine initiation

### Data extraction

Two reviewers (JO & PP) will independently extract data onto an identical data collation tool on Microsoft Excel. This will be a bespoke form designed prior to data collection, and will record patient demographics (age, sex, and ethnicity), pharmacotherapy details (clozapine titration regimen and concurrent medications), heart rate (baseline and peri-titration), other clinical signs and symptoms, and positive investigation results.

A third reviewer (DR) will adjudicate over any disagreements in data extraction between the two initial reviewers.

### Quality assessment

All articles meeting our inclusion criteria will be collated and summarised. The JBI checklist for case reports [37], items 1–6, will be used to determine the quality of case studies for inclusion in more detailed analysis, whilst the Newcastle-Ottawa quality assessment scale [38] will be used to determine the quality of case-control and cohort studies. Items 7 and 8 of the JBI checklist were excluded because an adverse event is inherent to all included cases given the focus on CICI (item 7), and the “takeaway lessons” item was not deemed to influence risk of bias or study merit (item 8).

Two reviewers (MM & MC) will review articles in their entirety before using either the JBI checklist or Newcastle-Ottawa scale (NOS) to independently allocate a rating to each article. Articles will be dichotomised into ‘good’ (high score) and ‘poor’ (low score) using a cut-off score of more than two-thirds (JBI checklist score of 4 or above, NOS score of 6 or above). In addition, all articles meeting our inclusion criteria will be rated in terms of quality using a GRADE quality rating. All articles will be placed into one of four levels, either ‘high’, ‘moderate’, ‘low’ or ‘very low’. Two reviewers (MM & MC) will independently review articles and allocate ratings accordingly. Articles will be presented in a ‘Summary of Findings’ table to guide the ultimate recommendations from the review. Any discrepancies or differences in opinion relating to the above assessments of quality and bias will be resolved by consensus and discussion with a third reviewer (DR).

### Data analysis

The lead reviewer (JO) will oversee all analyses to ensure methodological consistency. Descriptive statistics will be used to summarise the demographic and key variables within the data, including the proportion of cases involving tachycardia. Chi-square analysis will be used to compare differences in presentation based on gender and ethnicity. Data will be quantitively stored and processed using Microsoft Excel software [39], before applying multiple regression analysis through Stata software [40] to assess whether differences in both age and clozapine titration rate led to variability in terms of tachycardia severity being present by considering both absolute heart rate as well as increase from baseline. Correlation coefficients from regression analysis will be taken as effect sizes. It is hypothesised that a more rapid titration will increase the risk of developing CICI [10].

Categorisation based on quality assessment will be used to perform sub-set analysis to identify differences in presentation according to methodological rigour. This may identify trends in methodological limitations which will lead to recommendations for future research in this area. In addition, and if sufficient data is obtained, a funnel plot will be created to identify publication bias. However, it is noted that this may not be conducive to the inclusion of predominantly case-report literature. Cases will be excluded if relevant data for analysis is missing. If a sufficient dataset is not available to conduct meta-analytical approaches outlined, a narrative review of the literature will be conducted. Wider information about other signs, symptoms and investigations associated with CICI will be explored on a narrative basis with no formal analytical methods being employed. All statistical analysis will be exploratory in nature, recognising the expected heterogeneity and limited sample sizes within the available literature.

### Participant and public involvement and engagement (PPIE)

PPIE will be implemented in this review as a cross-sectional involvement activity where representatives with relevant lived experience will be asked to complete a questionnaire. PPIE representatives will be identified through existing clinical networks. The short online questionnaire will be centred around lived experience of the clozapine titration process, including the various investigations that are required during the initial weeks of treatment. It will explore whether patients would be open to additional investigations, such as pulse checks, electrocardiograms, and additional blood tests alongside mandatory full blood counts.

The questionnaire will ask for anonymised patient demographics, medication usage, any side-effects and changes to treatment. This questionnaire will be distributed to various mental health teams around West Yorkshire for distribution to their patients to complete online. Formal ethical approval will not be required for this PPIE activity [41], and all representatives will complete a clear consent statement prior to undertaking the questionnaire.

This consultative activity will contextualise the findings from this review, rather than influencing study selection or interpretation, ensuring that patient perspectives inform understanding of the results. We anticipate enlisting around ten to fifteen respondents to this questionnaire from the Yorkshire and Humber region.

### Ethics and dissemination

This review will use existing published data, and as such, formal ethical approval is not required. The review will be registered on the PROSPERO database prior to the database search commencing. Findings from this review will be published in a peer-reviewed journal, including a plain language summary for use in other literature. We will present our findings at national psychiatric and psychopharmacology conferences, as well as disseminating within mental health trusts and academic institutions through existing links to guide clinical practice.

## Discussion

This review intends to determine the clinical utility of heart rate monitoring during the initial stages of clozapine initiation. Optimising clinical use of clozapine is crucial, given its role as a highly effective antipsychotic medication which is only used when at least two other drugs have failed. By determining the most effective method of detecting its adverse effects, we intend to increase the safe prescription and monitoring of this effective and underutilised medication.

The strengths of this review include its novelty and broad search strategy, including clinical case reports and grey literature. However, this can also be seen as a limitation. The nature of case reports means that a thorough and comprehensive assessment of bias is needed to ensure that appropriate conclusions are made.

One additional aspect that may be considered a limitation to this proposed protocol is the chosen thresholds used to define tachycardia. In addition to 100bpm, we have included an increase of 30 beats per minute from baseline following clozapine initiation. This has been additionally included in order to capture patients who may have experienced a significant elevation in heart rate, but due to a lower baseline heart rate (e.g., 50–69bpm), were not considered as tachycardic under the standard definition of 100bpm. The threshold was adapted from the definition for postural orthostatic tachycardia [42].

The utility of a clinical tool depends on both sensitivity and specificity [43]. Unfortunately, due to the nature of published case reports, it is unlikely that this review will detect cases where CICI was suspected but then excluded. Therefore, it might be difficult to ascertain the specificity of tachycardia in detecting CICI through this review. This is of course a key limitation to this review, and in isolation cannot guide diagnostic clarity or utility, but only capture and amalgamate the sensitivity of tachycardia in detecting CICI from existing literature. From our scoping review, it appears that some studies have previously commented on the specificity of tachycardia in cardiac inflammation [27]. However, it is envisaged that the same accuracy will not be achieved with regards to specificity when compared to sensitivity, which will limit the application of our findings to determining the utility of tachycardia as a detecting tool.

Another limitation to our study design could be said to be the inclusion of people with pre-existing cardiac conditions such as coronary heart disease or heart failure. This may lead to misidentification of tachycardia as being solely clozapine-induced. However, where a case report or study lists in sufficient detail a patient’s baseline condition and changes to this that manifested after introduction of clozapine, we felt that this could still be of importance in literature, and therefore it would not be appropriate to exclude findings solely on the purpose of pre-existing conditions.

The nature of CICI is of a rare but serious adverse effect that is, at present, captured in academic literature predominantly through case report and case series literature. This introduces significant heterogeneity to the review process, which is of course a notable limitation. Due to the envisaged lack of wider-scale studies and an expected dependence upon case report literature, a formal heterogeneity assessment has not been incorporated as part of this protocol, which could be considered a limitation to this proposed literature review. However, a comprehensive narrative review of existing case literature is presently lacking, which is where this review is envisaged to be of benefit.

This review has focussed exclusively on CICI occurring during the initial titration period of clozapine, defined by the authors as within 45 days of commencing clozapine. As a result, late-onset myocarditis, which has occasionally been reported in literature [44], has been excluded. This criterion was established in order to assist this review’s aim to primarily ascertain the envisaged benefits of stringent heart rate monitoring during the titration period, which currently features in clinical policy [45].

The use of a diagnostic standard for our review was the detection of CICI through either biopsy or imaging techniques. We have excluded cases where diagnosis was made solely on clinical information alone. Whilst the use of multiple investigation techniques introduces a heterogeneous reference standard, this criteria was determined due to the limited use of cardiac MRI or endomyocardial biopsy being utilised in clinical practice, whilst still maintaining diagnostic confirmation through myocardial imaging.

As stated earlier, the clinical utility of this review in isolation is limited by its reliance upon case reports and focus upon sensitivity rather than specificity, and we acknowledge that sensitivity alone cannot establish clinical utility. While specificity cannot be determined within this review, synthesising sensitivity data will guide future work toward defining diagnostic parameters for CICI. We intend to use the findings from this systematic review to devise a prospective cohort study of clozapine titration, comparing development of tachycardia along with other clinical signs and symptoms with the onset of cardiac inflammation for various titration protocols. This will allow for greater understanding around the specificity of tachycardia as a predictive tool for CICI during clozapine initiation.

### Article Summary – Strengths and limitations

- Wide array of databases covered to maximise breadth of review
- Inclusion of case reports allows for greater scope of review, although open to greater degree of bias
- Review aims to establish sensitivity of tachycardia for CICI, but will be unlikely to determine specificity

## Supporting information

S1 Fig Preliminary search strategy for Ovid Medline. (DOCX)

S2 Fig Reporting checklist for protocol of a systematic review and meta analysis. (PDF)
