# Supplementary material for: Reliability of tachycardia as an early warning sign in acute clozapine-induced cardiac inflammation: Protocol for a systematic review
Source: PLoS One. 2026 Feb 25;21(2):e0341729. doi: 10.1371/journal.pone.0341729 (PMC12935219; doi:10.1371/journal.pone.0341729)
Supplement: S1 Fig — (DOCX) [file pone.0341729.s001.docx]

**Supporting Information 1: Preliminary search strategy for Ovid Medline**

Ovid MEDLINE(R) ALL <1946 to January 27, 2025>

1 clozapine/ 9758

2 (clozapine or denzapine or zaponex or clozaril or fazaclo or versacloz or clopine or synthon).mp. 17224

3 1 or 2 17224

4 Myocarditis/ 18064

5 (myocarditis or pericarditis or myopericarditis or perimyocarditis or carditis).tw,kf. 39076

6 Cardiotoxicity/ 5416

7 exp Heart/ 571495

8 (cardiac or heart).tw,kf. 1506561

9 exp pericarditis/ 12777

10 (cardiotoxic* or cardio-toxic* or cardiomyopath* or cardio-myopath*).tw,kf. 116357

11 Cardiomyopathies/ 35018

12 4 or 5 or 6 or 7 or 8 or 9 or 10 or 11 1790824

13 Heart Rate/ 179938

14 (heart adj4 (beat* or rate*)).tw,kf. 208966

15 exp Tachycardia/ 53273

16 (tachycardi* OR pulse).tw,kf. 276297

17 13 or 14 or 15 or 16 555666

18 exp Vital Signs/ 450666

19 vital sig*.tw,kf. 22870

20 18 or 19 469820

21 3 AND (12 or 17 or 20) 1005
